# Supplementary material for: Structural features of the streetscape of Macau across four different spatial scales based on historical maps
Source: PLoS One. 2021 Oct 12;16(10):e0258086. doi: 10.1371/journal.pone.0258086 (PMC8509972; doi:10.1371/journal.pone.0258086)
Supplement: S1 File — (DOCX) [file pone.0258086.s001.docx]

**Title：Structural Features of Streetscape in Macau at Four Different Spatial Scales, Based on Historical Maps，Information files**

**Link：https://figshare.com/s/b21949bb519df293fe51**
