# Supplementary material for: Structural features of the streetscape of Macau across four different spatial scales based on historical maps
Source: PLoS One. 2021 Oct 12;16(10):e0258086. doi: 10.1371/journal.pone.0258086 (PMC8509972; doi:10.1371/journal.pone.0258086)
Supplement: Attachment — Submitted filename: Response to Reviewers.docx [file pone.0258086.s002.docx]

Dear editor,

Thank you very much for your decision letter and additional advice on our manuscript (Manuscript # PONE-D-20-36948R1) entitled “Structural Features of Streetscape in Macau at Four Different Spatial Scales, Based on Historical Maps”. We also thank the reviewers for their review of our revised manuscript and further comments. We are pleased to have the opportunity to address their additional concerns, and as before, all amendments are indicated by red font in the revised manuscript. In addition, our point-by-point responses to the latest comments are listed below this letter.

We hope that this further revised draft of the manuscript is now acceptable for publication in your journal and look forward to hearing from you soon.

With best wishes,

Yours sincerely,

Shuai Yang

First of all, we would like to express our sincere gratitude to the reviewers for their constructive and positive comments.

**Replies to Reviewer 1**

Reviewer #1: General Comments
The authors made no attempt to make their case as to why this paper should be published. The abstract and introduction do not explain why the research matters. They do not describe the literature gap or main research question, nor do they put the research in context with other previous work. Both should be rewritten.

**According to your opinion and the following detailed suggestions, this part has been explained in detail in the Abstract and Introduction. In the revision part, the author added questions such as why this paper should be published, why the research matters, and describe the main research question**

**The Abstract and Introduction have been rewritten.**

In addition to a rewrite of the abstract and intro, the paper would benefit from an intensive edit for structure, flow, and clarity（. Specifically, the methods and results sections need to be reordered and restructured. Results need to be rewritten to focus on the main findings, which are confused by having too many figures. Discussion describes findings that were not presented in the results section. Intro and conclusions should mirror each other in structure.

**I also found such problems in the revision process. And I have made great adjustments and modifications to the structure and content of the article according to the suggestions, which can be shown in the revised article.**

The authors included no line numbers or page numbers, which made the review difficult. I made suggestions for structure that would improve the manuscript; I do not point to specific text that needs changes.

**Sorry for the trouble caused to the Reviewer. Line Numbers and Page Numbers have been added**

I could not find info about data deposition in the manuscript. Given that the authors produced shapefiles from four different time periods and calculated intelligibility for each time period, I would expect a substantial amount of geospatial data was produced. What are the plans for depositing the data?

**In this paper, all spatial objects in the spatial syntactic analysis software are modeled based on SSOODM, formed axis map to calculate free spatial morphological variables, and stored on ArcGIS platform. The intelligibility index was obtained after running the syntax software AXWOMAN 6.3. The data of local integration degree and global integration degree are imported into Excel, and the correlation analysis between the determination coefficients of the fitting line is made. Finally, the comprehensibility of the four historical periods is plotted and the analysis results are obtained.**

**Abstract**
The abstract does not provide enough detail explaining study. Consider writing five sentences that will set the structure for the rest of the paper.

1: What do we know?

**In 2011, UNESCO pointed out in "Recommendations on Urban Historical Landscapes" that "The historical urban landscape is regarded as the geographical entity of the urban area, and it is understood as the result of the historical accumulation layering f various values."**
2: What we don't know?

**Although the official put forward the innovative significance and concept of historical layering, there are not too many reference cases when implemented at the operational level. For example, when it is necessary to analyze the complex historical layering, there is less research on how to get rid of theoretical analysis and use quantitative forms to display and analyze.**
3: How did you fill knowledge gap described in sentence 2?

**The space syntax used in this paper fills the gap of quantitative analysis in historical layering analysis. The article establishes a spatial digital model by combining the urban historical landscape theory with the method analysis of space syntax. A quantitative analysis of the Streetscape in the four historical periods of Macau, and the value-related development of the city's economy, society, and culture. The article uses the theory of urban historical landscape to interpret the Streetscape of Macau.**
4: What did you find out?

**Urban development is reviewed under different Spatial Scales, presenting different historical layering states. The changing ideological trend of construction brings about the change of the city, which leads to the change of the city style. Its identifiability is constantly changing with the time layering.**
5: Importance of your work, broader implications, future research

**In the process of analyzing the urban historical landscape, this paper proposes a novel analysis method, which is to use space syntax to quantitatively analyze its spatial form and momentum. The analysis has both the dimensions of space and time, and its results have played an important role in how to continue the life course of the benign growth of the urban landscape and solve the protection problems in practice. At the same time, to provide reference for the protection of historic cities, there is still a lot of work to be done in-depth and unfolded for the future in-depth analysis of more complex historic cities.** **Through the research of this article, the value of Macau's historical streetscape can be retrieved, and the targeted protection strategy can be implemented so that the historical streetscape of Macau can be passed on.**

Based on abstract, I don't know why the urban streetscape is important or what its historical value is. Authors say their results deeply explore Macau streetscape but do not describe those results or put them in a broader context.

**As an important part of the regional characteristics of Macao, the urban streetscape of Macao is the continuation of the historical memory of Macao and has irreplaceable historical value. In the context of urban development, the ancient streets always have a unique charm in Macau, such as the “straight streets” of Macau. However, with the advancement of this research, it is found that the unique streetscape of Macau bred by the development of the city does not affect the newly constructed roads. The newly reclaimed area and the streetscape of the new city are on the verge of homogeneity, and basically cannot reflect the unique regional characteristics of Macau. Therefore, Through the research of this article, the value of Macau's historical streetscape can be retrieved, and the targeted protection strategy can be implemented so that the historical streetscape of Macau can be passed on.**

**Introduction**
The intro does not provide enough detail to make a compelling case for the study. Why is layering important? Why should you use spatial syntax theory? What are the alternatives? What literature gap are you filling? What are the contributions of your paper?

**The United Nations Educational, Scientific and Cultural Organization (UNESCO) published an instructional collection of essays in 2017 detailing the importance of historical layering for the study of historic urban landscapes，and conducted a detailed case study of historical layering in Cuenca, Ecuador. Macau and Cuenca have many similarities since their initial rise. Both of them used to be colonies, and under the continuous action of time and space, both of them are continuously taking place historical layering process. Urban space or maintain, or replace, or decline, or at the same time, the formation of stratification and juxtaposition as the main situation. The study of UNESCO provides an important theoretical basis for the study of the historical layering of Macao.**

Why is layering important?

**The law of urban evolution in the study of historical layering contains the great wisdom of predecessors, and provides a clear context for urban development. And the experience and lessons contained in the study provide valuable reference for the development of modern cities. The wisdom of our ancestors, such as cultural tradition, construction technology, morality and etiquette, is worth inheriting and carrying forward. At the same time, it also provides some positive countermeasures for the future development of the city.**

Why should you use spatial syntax theory?
**The introduction of space syntax theory is helpful to analyze data quantitatively. The spatial syntax is used to analyze the street and lane network in GIS environment. The very close interaction between spatial form and human behavior is revealed in a scientific way, and the relative importance of each space in the whole spatial system can be easily studied by analyzing data and images. It is a more intuitive and quantitative research method and reflects the form and development of streetscape in this historical period through various syntactic indicators.**

What are the alternatives

**In the aspect of methodological research on urban historical streetscapes, in addition to space syntax, Kevin. Lynch, Gordon Cullen and other scholars adopted the method of base analysis and made outstanding contributions to the analysis and research of SITE. In his book Site Planning, Lynch put forward a set of Site analysis methods involving social, cultural, psychological, natural and material elements of the context, which has had a significant impact on modern urban design. Cognltive Map is also an effective method to study urban historical streetscapes, which is an urban spatial analysis technology borrowed from the field of cognitive psychology. The concrete process also uses the sociological survey method for reference, which is an effective way to control the urban landscape and place image. The specific approach is to investigate the residents' psychological feelings and impressions of the city through inquiry or written methods, and then let the designers analyze and make them into maps, or more directly encourage them to draw sketches of the spatial structure of the city. In addition, drawing "landscape narrative historical map" is another method, which expresses the changing process of the landscape in a certain region through the presentation form of the map. This method has taken Shanghai as an example to elaborate on the making of its historical and cultural maps.**

What literature gap are you filling?

**This paper combines the ancient map of Macao with space syntax to explore the quantitative research methods of layering in different historical periods and their applications.**

What are the contributions of your paper?

**The concept and method of urban historical landscape give us a new perspective to understand and recognize the layering change of the whole city. The main purpose of this paper is to arouse people's cognition of urban historical and cultural heritage.**

The introduction is two paragraphs and superficially describes layering. I expected to see more urban morphology or time and space urban literature.

Lynch K. What time is this place?. MIT Press; 1972
Matos Wunderlich FI. Walking and rhythmicity: Sensing urban space. Journal of Urban Design. 2008 Feb 1;13(1):125-39.
Sheng N, Tang UW, Grydehøj A. Urban morphology and urban fragmentation in Macau, China: island city development in the Pearl River Delta megacity region. Island Studies Journal. 2017 Nov 1;12(2):199-212.
Chung T. Valuing heritage in Macau: On contexts and processes of urban conservation. Journal of Current Chinese Affairs. 2009 Mar;38(1):129-60.
Griffiths S, Jones CE, Vaughan L, Haklay M. The persistence of suburban centres in Greater London: combining Conzenian and space syntax approaches. Urban Morphology. 2010;14(2):85-99.

**Thank you very much for the comments of Reviewer. I have read all the above papers carefully and added relevant important contents in my paper, especially the contents of Urban morphology and urban space type of Macao. These are also added in the references.**

A 5-7 paragraph intro structure to consider:
P1: This is important idea but there are problems with it
P2: Problems/challenges with it are these (x,y,z)
P3: Implications of paragraph 2
P4: Here is how we approach the problem
P5: Contributions of paper and its structure
(you may need up to 2 more paragraphs)

**I have made some modifications according to the suggestion, and the paragraphs of introduction has been extended to 4**

**Materials and Methods**
This section needs to be reordered. Space syntax theory needs to be in the introduction. Then, the authors need to explain how they collected data, processed it, and analyzed using the specific space syntax methods implemented in the study. Look at Ma et al. 2020 [13] as an example for how you could structure this section in addition to my comments below.

**The article is already being reordered. Space syntax theory has been adjusted to the introduction section. For collected data and processed, the author selected the historical maps of 1780, 1889, 1950 and 2018 as the base map for layering analysis and self-drawing. The base maps (1780, 1889, 1950) come from Cultural Affairs Bureau of the Macao S.A.R. Government. The Archives of Macao. The base map (2018) comes from USGS National Map Viewer. https://viewer.nationalmap.gov/advanced-viewer/. First of all, the four times street and lane structure drawings are obtained by using CAD. Then, according to the drawings, the integration degree and crossing degree of the street and lane structure of the four time are derived by using GIS and space syntax, and then the stratification status of the historical streetscape of Macao in different periods is obtained.**

Authors say that intelligibility is a correlation and don't say what kind of correlation (Pearson?). Figures 17-20 are plots of intelligibility (overall integration on y axis and local integration on x axis) and have a regression line. Figure 21 plots intelligibility coefficients for each time period.
**Correlation is used to describe the degree of correlation between the local control and the overall association. It reflects how easy it is for the subject to distinguish the local space pattern from the overall space pattern. Local space and global space must exist, and there is a certain degree of correlation. The greater the correlation is, the more consistent the local perceptible space is with the overall invisible space, so the spatial form is easier to be understood.**

**The Numerical analysis of the intelligibility in Figures 17-20 gives a detailed explanation of the process. R2 is explained and the calculation formula is added.**

$${R^{2}=\frac{\left[ \Sigma\left( C_{i}-\bar{c} \right)\left( I_{i}-\bar{I} \right) \right]}{\Sigma\left( C_{i}-\bar{c} \right)^{2}\Sigma\left( I_{i}-I \right)^{2}}}^{2}$$

I believe that the authors performed regression analysis, and I want the models summarized in a table. Adjusted R2 for model plus coefficients for all variables in models.
**The models have summarized in a table, and I have added the content to the appropriate location as requested.**

I found no description of possible error sources.
**It has been added in Section 2.3**
The last two paragraphs of the section still contain material from a template (language regarding datasets and animal care). This should be removed.
**These two paragraphs have been removed.**

Consider the following structure:
2.1 Study Area - More detail describing Macau. Authors assumed all readers have familiarity with study area, which will not be true. Include some brief historical context covering your time periods of interest: governance, population, economics, social change, etc.
**The Space Syntax section has been moved up to Introduction. From the perspective of society, culture, architecture and so on, more detail about Macau is added. It emphasizes that Macao used to be a colonialist country, and its multi-coexistence and mutual integration of social culture is an important social feature.**
2.2 Data Acquisition and Processing
Include all details of how you obtained data and processed it. Do not include any analysis here. Something like this:
We obtained official historical maps for the years _____, ____, ___, ___ [data sources]. Using ARCGIS (version?) and CAD (version?), we vectorized each map (how specifically?).
**It has been carefully modified according to the requirements.**

2.3 Data Analysis
Only include data analysis here:
"We imported the calibrated data into Depthmap (source, version?). For each time period, we calculated the following spatial syntax variables (name them all.) Then, discuss how you did the spatial syntax analysis specifically. Explain how you calculated each element, using the formulas for angle integration, angle choice, intelligibility etc. Provide citations for each formula. “

**This part of the content proposed by the Author has been introduced in detail in the original 2.1 section on space syntax, which has been slightly modified into Section 2.3. The Depthmap version was depthmapX-0.7.0. Adding and refining the formula for the calculation, and Providing citations for each formula.**

Results and Discussion
Results are unfocused, because authors have too many figures. Some of the results are included in the discussion as well. If the authors are going to have separate results and discussion sections, then results need to be concisely presented.
**Results and Discussion have been merged into one chapter. At the same time, I have revised about the content and structure to make my results look focused.**

Then, in discussion, interpret the results specifically for Macau context, and then compare to other studies that have applied similar techniques. The authors cite 5 sources in the discussion: 4 relating to Macau and 1 to Naples, Italy.
Discussion brings in some historical context (4 references), but for a reader not familiar with Macau context, it is not easy to link that context with specific results.

**“Macao is the unique witness of the first contact and collision between China and the West, which lasted for a long time. From the 16th to the 20th centuries, Macao was a meeting point for merchants, missionaries, and different fields of learning. This meeting of different cultures can be traced in the region represented by the core of Macao's history. Macau is an outstanding representative of the architectural community, which exemplifies the collision and development of Chinese and Western civilization in four and a half centuries. Macau is represented by a series of Spaces and architectural groups that connect ancient Chinese ports to Portuguese cities in the historical route. On July 15, 2005, the historic district of Macao met the selection criteria and was inscribed on the World Heritage List, making it the 31st World Heritage Site in China. The street landscape of Macao inherits the characteristics of the integration of Chinese and western cultures in Macao, which is deepened in the process of stratification and becomes an important feature of the city.” The content of Macao has been added in Discussion.**

The last line of the discussion (below) contains the reference to Naples [26], but it is not clear whether/how/if the Neapolitan context applies.
" Therefore, from the syntactic analysis data, the author examines the characteristics of the urban structure under the impact of such multiculturalism. [26]."
**There is an error here and the reference has been deleted**

Conclusions
The conclusion should mirror the structure of the intro. Succinctly explain what you did, briefly describe your findings, and their broader implications. What are some future research directions? What are the planning implications of your work?
**It has been modified according to the requirements**

Figures in General
Too many figures, so the story is lost. I recommend reducing the total number of figures to 6 maximum to best show the patterns of change and to focus the narrative of the paper. Incorporate source of image or analysis into caption.

**This article has reduced a lot of unimportant images. It has been noted in the revised article. However, a few pictures were very important, so they were not deleted. There were 1-2 more pictures than the review expected.**

Consider combining some of your figures so that the trends across each time period are more obvious. Strengthen introduction and putting your results in context of Macau and other cities where others have applied these methods,
**Thanks for the comments, I have rewrote the introduction and results.**

Figures specifically:
1: Move to supp info. Change caption (e.g., Example structural features of streetscape in Macau at three different spatial scales, based on 2019 map (cite Google maps). Then, label scale on top (assuming they are zoomed in to same spatial scale for each box).
**Caption is modified to “Structural Features of Streetscape in Macau at Four Different Spatial Scales, Based on maps (1780\1889\1950\ 2018).”**

2: Keep in main paper. Historical layering of straight streets in Macau, based on maps from 1796, 1889, 1953, and 2010 [data source].
**It has been kept in main paper. However, considering that some pictures can’t be authorized, we choose pictures (can be authorized) that have a similar year instead.**

3-4, 7,8, 10,11,13. Delete or move to supplementary info. These maps are source data for your analysis.

**The images have since been deleted**
5,9,12,14,15 - Keep. These are your analysis results. Interpret these.
**Fig. 5,9,12,14,15 are kept. As Fig.5, 8, 11 and 13 are all the structural drawings of streets and alleys in Macao (in different historical periods), for the article integrity, Fig.5 and 6 are combined into one picture, Fig.8 and 9 are combined into one picture, Fig.11 and 12 are combined into one picture, and Fig.13 and 14 are combined into one picture.**
15 - Excellent figure. Describe in results; interpret in discussion.

**The location of Figure 15 has been changed. And made a supplement according to the request.**
17-21. Can these be combined into a four panel figure for each time period, with the coefficient displayed in the upper right?
**It has been modified as required**

References - authors only cite 26 sources. More are needed.
**On the basis of the original, added more References, there are nearly 50 References**

Reviewer #2: 1. The “Abstract” section should include background, methods, results and conclusions. The background should be concise 1-2sentences, and the results should be the major findings of your study section. Please modify this section accordingly.

**Regarding the Abstract part, Reviewer 1 also puts forward corresponding suggestions. Based on the opinions of two Reviewers, the Abstract has been rewritten.**
2. The current “1. Introduction” section mainly introduces the concept of historical layering. Authors are highly encouraged to provide the relevant background knowledge necessary for the readers to understand why the findings of the paper are an advance on the knowledge in the field. You also need cited more new references, and give the research gap more clearly.

**The "Introduction" section has been rewritten according to the requirements, and more references have been added to expand a large part of the knowledge. The "Introduction" section is not only about historical layering, but also about urban historical landscape, space syntax, domestic and foreign research status of this part, etc., to highlight the findings of the paper are an advance on the knowledge in the field.**
3. The “2. Materials and Methods” section introduced space syntax, but it is not explained why the spatial syntax is used to study the historical layering of urban streetscape in Macau. It is recommended to explain it in the introduction or the methods section.

**About "Why the spatial syntax is used to study the historical layering of urban streetscape in Macau." I have adjusted the structure of the article, explicitly and in detail added this content in "Introduction", and also put forward other alternative methods.**

4. The “4. Discussion" needs to be improved, because it presents only discussion of the results obtained, without correlating with the scientific literature, thus, demonstrate the importance of the results obtained. Besides, the analysis on intelligibility of historical maps of streetscape in Macau should be moved to the results section and not fit into the discussion.

**The structure of the article has been modified and relevant content added as suggested by Reviewer #2.**
5. In the “5. Conclusions” section, the statement that the names of streets are a major feature of the historical landscape of Macau is a new knowledge, which has little to do with the subject of the article. It is recommended that the main findings be stated in conclusions.

**The "The Names of Streets" section has been removed as requested. At the same time, the conclusions are revised and the main findings are added**.

6. PLOS authors have the option to publish the peer review history of their article ([what does this mean?](https://journals.plos.org/plosone/s/editorial-and-peer-review-process#loc-peer-review-history)). If published, this will include your full peer review and any attached files.


If you choose “no”, your identity will remain anonymous but your review may still be made public.


**Do you want your identity to be public for this peer review?** For information about this choice, including consent withdrawal, please see our [Privacy Policy](https://www.plos.org/privacy-policy).

Reviewer #1: No

Reviewer #2: No

[NOTE: If reviewer comments were submitted as an attachment file, they will be attached to this email and accessible via the submission site. Please log into your account, locate the manuscript record, and check for the action link "View Attachments". If this link does not appear, there are no attachment files.]

While revising your submission, please upload your figure files to the Preflight Analysis and Conversion Engine (PACE) digital diagnostic tool, <https://pacev2.apexcovantage.com/>. PACE helps ensure that figures meet PLOS requirements. To use PACE, you must first register as a user. Registration is free. Then, login and navigate to the UPLOAD tab, where you will find detailed instructions on how to use the tool. If you encounter any issues or have any questions when using PACE, please email PLOS at [figures@plos.org](mailto:figures@plos.org). Please note that Supporting Information files do not need this step.
